# Comparative Study of Transcriptome Profiles of Mechanical- and Skin-Transformed *Schistosoma mansoni* Schistosomula

**DOI:** 10.1371/journal.pntd.0002091

**Published:** 2013-03-14

**Authors:** Anna V. Protasio, David W. Dunne, Matthew Berriman

**Affiliations:** 1 Wellcome Trust Sanger Institute, Wellcome Trust Genome Campus, Hinxton, United Kingdom; 2 Department of Pathology, University of Cambridge, Cambridge, United Kingdom; McGill University, Canada

## Abstract

Schistosome infection begins with the penetration of cercariae through healthy unbroken host skin. This process leads to the transformation of the free-living larvae into obligate parasites called schistosomula. This irreversible transformation, which occurs in as little as two hours, involves casting the cercaria tail and complete remodelling of the surface membrane. At this stage, parasites are vulnerable to host immune attack and oxidative stress. Consequently, the mechanisms by which the parasite recognises and swiftly adapts to the human host are still the subject of many studies, especially in the context of development of intervention strategies against schistosomiasis infection. Because obtaining enough material from *in vivo* infections is not always feasible for such studies, the transformation process is often mimicked in the laboratory by application of shear pressure to a cercarial sample resulting in mechanically transformed (MT) schistosomula. These parasites share remarkable morphological and biochemical similarity to the naturally transformed counterparts and have been considered a good proxy for parasites undergoing natural infection. Relying on this equivalency, MT schistosomula have been used almost exclusively in high-throughput studies of gene expression, identification of drug targets and identification of effective drugs against schistosomes. However, the transcriptional equivalency between skin-transformed (ST) and MT schistosomula has never been proven. In our approach to compare these two types of schistosomula preparations and to explore differences in gene expression triggered by the presence of a skin barrier, we performed RNA-seq transcriptome profiling of ST and MT schistosomula at 24 hours post transformation. We report that these two very distinct schistosomula preparations differ only in the expression of 38 genes (out of ∼11,000), providing convincing evidence to resolve the skin vs. mechanical long-lasting controversy.

## Author Summary

Schistosomiasis is an endemic parasitic disease affecting ∼200 million people in the most socioeconomically deprived regions of the world. Human infection occurs during water contact where free-living larvae called cercariae penetrate host skin and become parasitic organisms called schistosomula. This stage represents the first encounter of the parasites with the host and is also regarded as one of the most vulnerable stages of the parasite's life cycle. Therefore, schistosomula are the focus of many studies, many of which look at changes in the expression of genes as a way of understanding the process of infection, identifying potential drug targets and vaccine candidates. Because collecting enough parasitic material from natural infections is not possible for certain types of studies (for example, gene expression studies), a mechanical transformation of the cercariae into schistosomula is often used instead and assumed as a good proxy for the natural transformation process. However, the equivalency of gene expression profiles between naturally transformed parasites and the mechanically transformed counterparts has never been studied. In this report, we analyse differences in gene expression patterns between these two different parasite preparations and provide enough data to resolve a long-lasting controversy.

## Introduction

Schistosomiasis is a parasitic disease caused by platyhelminths of the genus *Schistosoma*. It has been estimated that ∼200 million people are infected and ∼200,000 die due to schistosomiasis-related pathologies [1]. Without a vaccine, mechanisms of prophylaxis rely primarily on reduction of the number of infected individuals through mass-administration of the only available drug praziquantel. However, the number of infected people has changed little over the last decades [1]. What is more, reduced susceptibility of *Schistosoma mansoni* worms to praziquantel has been reported in the field [2], [3] and resistance to the drug can be induced under experimental conditions [4], [5], raising the possibility that a similar situation could be also seen in the field. Consequently, the development of new mechanisms of intervention is a priority.

In this context, it is important that the process of infection is well characterised. The infectious agents for the human host, the cercariae, are microscopic free-living larvae released by infected fresh water snail hosts. Cercariae infect their mammalian host during water contact by trespassing across the skin barrier. This process is characterised by rapid morphologic, metabolic and physiological changes [6]–[9] that results in obligate parasitic schistosomula in as little as 2 hours [10]. The most prominent aspects of this transformation are the loss of the cercarial tail and a series of changes in the parasite's surface. During skin penetration, the outermost layer in the parasite's surface, the glycocalyx, gets thinner by the action of secretions from the parasite's own acetabular glands [11], which are emptied during the process of transformation [12]. The remains of the glycocalyx are shed together with transient microvilli structures that form and disappear during this transformation process [13]. At the same time, pre-packed multi-laminated vesicles originating from the body of the parasite make their way to the surface where they release their contents; these contribute to the generation of the new double-bilayer membrane, characteristic of the intra-mammalian stage of the parasite [14].

Increasing research on the schistosomulum stage required the development of efficient, reproducible and rapid ways to generate large quantities of biological material. Various effectors are known to elicit the artificial transformation of cercariae into schistosomula, for example, cell growth media at 37°C [15], [16] or just low osmolarity phosphate buffer saline solution [17] seem to be enough to trigger the cercariae to schistosomula transformation. The presence of certain skin lipids, yet is not essential [17], also plays a role in the process of cercariae transformation and penetration [18], [19] probably by triggering the release of acetabular glands contents [20]. The most popular method for obtaining artificially transformed schistosomula uses a mechanical transformation (MT) protocol that includes some sort of shear force (centrifugation [21]–[23], passages through an emulsifying needle [24], or shaking [15]) applied to freshly shed cercariae followed by separation of cercariae heads from tails (usually by centrifugation in a density gradient) and posterior incubation of the cercariae heads/schistosomula in culture media at 37°C. Parasites obtained using this protocol show no major morphological or biochemical differences with those recovered from natural infections [10], [15]; making the MT the method of choice for obtaining large quantities of schistosomula.

However, at the level of the whole transcriptome, equivalency of MT schistosomula to those obtained from natural infections has not been established; even though these artificial parasite preparations have been used almost exclusively in the identification of potential vaccine proteins and in high-throughput studies of gene expression [25]–[29], identification of drug targets [27] and screening of a compound library [30]. Artificial induction of stress or mechanical damage may induce gene expression signals that are not responding to the natural process of infection. Moreover, failure to induce physiologically important transcription events, triggered by host-skin specific signals, could lead to exploitable vulnerabilities being missed. Our work presented here uses high throughput transcriptome sequencing technology, known as RNA-seq [31], in combination with the latest genome assembly available for *S. mansoni* [32] to compare the profile of genes expressed in MT and ST schistosomula.

## Materials and Methods

### Biological material

*S. mansoni* (NMRI strain of Puerto Rican origin) cercariae were shed from infected *Biomphalaria glabrata* snails by exposing them to the light for 1.5 hours. MT schistosomula were obtained using an optimised version of the protocol used by Brink *et al.*, [10]. Optimisation steps of the protocol were implemented in the tail detachment step (shake cercariae vigorously for approximately 30 seconds in a vortex mixer before passing these through a 21G syringe needle approximately 13–15 times) and the separation of heads/schistosomula and tails (by placing the heads plus tails suspension on 10 ml of ice-cold 70% Percoll (Sigma, UK) and 90 mM NaCl solution in DMEM in 15 ml conical tubes) by centrifugation at no more than 1000 g for 10 minutes at 4°C.

**Figure 1:**
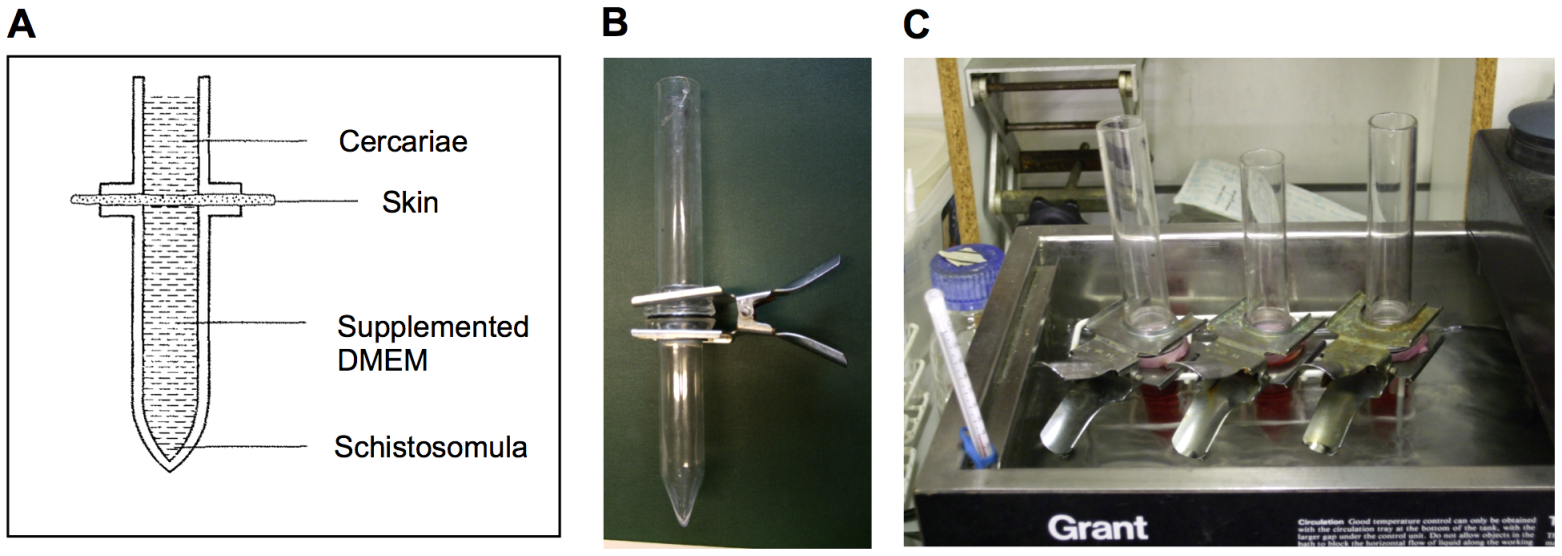
Diagram and photographs of assemblies used in skin-transformation of schistosomula. A – Graphical representation of a transformation assembly. B – Photograph of one of the transformation assembly prior to use. C – Three transformation assemblies in use during an experiment. The lower compartment of the assembly is placed in a water bath with a constant temperature of 37°C while the upper compartment is left at a room temperature (28°C).

Skin-transformed (ST) schistosomula were obtained using a modified version of the protocol published by Clegg *et al.*, [33]. TO (Tuck Ordinary) mice (Harland, UK) were killed with an overdose of anaesthetics followed by cervical dislocation according to Home Office regulations. Hair was removed from the abdominal and dorsal skin areas using clippers and skin was later excided from the animal using dissecting scissors. Each animal provided an area of skin of approximately 7.5 cm^2^; which was divided into two halves. Gel-like dermal tissue was removed by rubbing the skin gently (for approximately 5 minutes) with sterilized gauze soaked in supplemented DMEM (Dulbecco's Modified Eagle's medium supplemented with 100 U/L penicillin, 0.1 mg/L streptomycin and 10 mM L-glutamine). The transformation apparatus is presented in Figure 1A. The lower compartment of the assembly was filled with supplemented DMEM containing 2% fetal calf serum (FCS) and one half of prepared skin was mounted covering the opening of the tube with the dermal side facing downwards. The upper compartment was placed above the lower compartment with a rubber O-ring in between. All pieces were kept in place by holding both tubes with a metal clip (Figure 1B). The skin surface was washed three times with aquarium water and assemblies were checked for leaks. All assemblies were placed in a water bath pre-warmed at 37°C; the bottom compartment of the assembly was constantly kept at this temperature (Figure 1C). Experiments were carried out in a room with controlled temperature of 28°C. Approximately 12,000–14,000 freshly shed cercariae kept in aquarium water were placed in each assembly and these were left in the water bath for 3 hours. Schistosomula preparations were individually checked for contamination with tails. Samples with more than 4% tails/cercariae contamination were discarded.

MT and ST schistosomula preparations were placed in individual tubes, washed 3 times in supplemented DMEM and incubated at 37°C and 5% CO_2_ for a total of 24 hours in growth media (supplemented DMEM, 10% FCS, 1% Hepes buffer). Schistosomula preparations were observed under the microscope using a Leica DM 1 L inverted microscope (Leica, Milton Keynes, Bucks, UK) at 10× or 40×. Video recordings were taken using a Dino-Lite AM-423X camera and DinoXcope software (Version 1.7.3). The criterion used for evaluating parasites is the one used in Mansour *et al.*, [34].

After the incubation period was completed, parasites were transferred to 15 ml conical tubes and centrifuged at 1,000 g for 5 minutes, supernatant was discarded and schistosomula were suspended in 1 ml of TRIzol reagent (Invitrogen, UK) and stored at −80°C until RNA extraction.

### RNA extraction, library preparation and sequencing

Total RNA from parasite material was extracted using TRIzol (Invitrogen, UK) according to manufacturer specifications. After extraction, RNA quality was assessed using an Agilent RNA 6000 Nano - Bioanalyzer and quantified using a NanoDrop ND-1000 UV-Vis spectrophotometer. RNA-seq libraries were prepared as previously described [31] and sequenced as 76-base paired reads using the Illumina Genome Analyzer IIx platform. Raw sequence data were submitted to ArrayExpress (http://www.ebi.ac.uk/arrayexpress/) under accession number E-MTAB-451.

### RNA-seq reads alignment and gene expression analysis

RNA-seq reads were aligned to the latest *S. mansoni* reference genome (version 5.0, [32]) using TopHat [35] (version 1.3.1) with default parameters except for minimum and maximum intron sizes which were set to 10 and 30,000 bp respectively. Other parameters that were specified included the type of library sequenced (set to standard cDNA Illumina library; –library-type fr-unstranded) and the mate pair distance (or insert size; -r option), which was calculated individually for each library. Only uniquely mapping reads were kept for further analyses. The number of reads aligned to each transcript was calculated using BEDTools [36] and used to calculate RPKM (reads per kilobase per million of reads mapped) values [31] for each transcript. A threshold RPKM value was calculated as described in Protasio *et al.*, [32] and transcripts with expression <2 RPKM were removed from the dataset resulting in the reduction of the total number of transcripts from 11,778 to 9,291 (2,487 transcripts had negligible expression in both samples). Differential expression of transcripts was performed using EdgeR [37]. P-values were adjusted for multiple testing [38] and the threshold for significance set at adjusted p-value≤0.05. A complete list with fold change values and associated adjusted p-values obtained from EdgeR are provided in Supplementary Table S1.

### RT-qPCR validation of differentially expressed genes

Relative expression of a subset of genes found differentially expressed between the ST and MT schistosomula were assayed using real time quantitative PCR (RT-qPCR). Primers for these genes were designed using Primer3 software [39] and ordered from Sigma, UK (primer sequences are available upon request). First strand cDNA was synthesised from 1 ug of original total RNA samples (MT2 and ST2 – Table 1) using SuperscriptII (Invitrogen, UK) according to manufacturers instruction. All RT-qPCR reactions were performed in a Mx3005P QPCR System (Agilent Technologies) and using KAPA SYBR FAST qPCR Kit (Kapa Biosystems). PCR efficiencies for each primer pair were calculated using 10-fold dilutions of MT2 cDNA. Relative gene expression for a given gene was quantified relative to the expression of a reference gene (rRNA18S). Cycle thresholds (Ct) for each reaction where obtained using the MxPRO QPCR Software (Agilent Technologies) and used in the Pfaffl equation [40] to calculate the fold change expression of a target gene between samples. Fold change values reported are the mean of four replicates. In order to compare RT-qPCR and RNA-seq derived fold change values, RNA-seq standard deviation (SD) was calculated using the method described by Busby *et al.*, [41].

**Table 1:** Summary of sequenced samples and alignment to the *S. mansoni* reference genome.

Sample	Replicate	Lane_id	Sequenced reads	Total reads mapped
MT 1	1	4912_5	50,616,612	61.80%
MT 2	2	4912_6	50,441,286	57.87%
ST 1	1	4912_7	44,496,358	53.13%
ST 2	2	4912_8	48,970,182	57.50%
			Mean	57.57%

MT: mechanically transformed; ST: skin-transformed.

### Metabolic activity of schistosomula

AlamarBlue incorporates a colour indicator of metabolic activity of the mitochondrial function [42] and has previously been used to assess the viability of schistosomula [34]. In order to assess metabolic activity of MT and ST parasites, 250 24-hours-old-schistosomula obtained from MT and ST methods were incubated in AlamarBlue (Invitrogen, UK) for either 3 or 24 hours prior to measurement. Eleven and 12 samples were assayed for MT and ST respectively. Absorbance was measured at 570 nm (with reference at 600 nm) using a microplate reader BioTek PowerWave HT (BioTek Instruments Inc., Winooski, VT, USA); data collection was performed using the software Gen5 (BioTek Instruments Inc., Winooski, VT, USA). Raw absorbance data is presented in Supplementary Table S2. Student's *t*-test was used to evaluate the significance between mean absorbance.

### Ethics statement

All animal procedures were performed in accordance with the UK Animals (Scientific Procedures) Act 1986 and as authorised on personal and project licences issued by the UK Home Office.

## Results

### Optimization of transformation protocols

**Figure 2:**
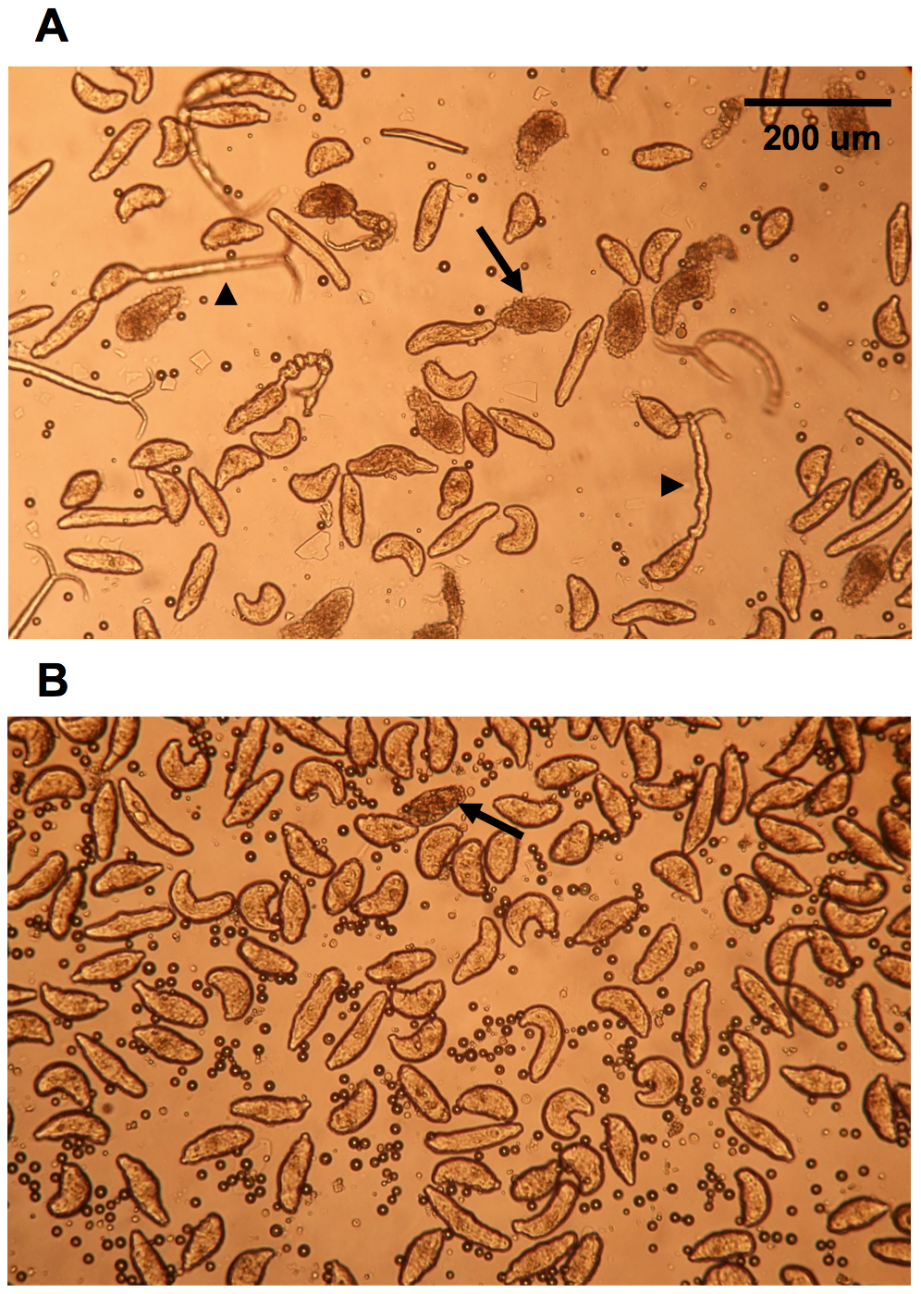
Improving the mechanical transformation protocol. A – Parasites were transformed according to a standard protocol [10] where parasites are subjected to 23 passages through a syringe needle and separation of heads and tails is performed with 60% Percoll solution at room temperature. B – Transformation of parasites was performed using the optimized protocol described in Material and Methods; which involved shaking of the cercariae suspension in a vortex mixer followed by only 12–13 passages through a syringe needle. Separation of schistosomula/heads from tails was performed using 70% ice-cold Percoll solution. Arrows indicate damaged schistosomula, arrowheads point to contaminating cercariae or tails. Both panels (A and B) show light microscope images of 3-hours post-transformation schistosomula.

Both MT and ST transformation protocols were subjected to optimization. Results comparing the standard and optimised MT protocols are shown in Figure 2A and 2B respectively. For the MT, optimised conditions resulted in increased numbers of schistosomula and a lower percentage of damaged or non-viable individuals. Reduction of the number of syringe passages resulted in increased number of viable parasites while a higher percentage of Percoll resulted in less contaminating tails (∼1%). For the ST, we found that schistosomula preparations virtually free from tail contamination (∼1% to 4%) could be obtained by placing no more than 14,000 cercariae in the upper compartment of the transformation apparatus. However, tail contamination was more frequent in the ST preparations than in MT and samples dedicated for RNA-seq libraries had to be carefully selected. Contrary to schistosomula resulting from MT, non-viable parasites were hardly ever observed in ST preparations. Under the light microscope, schistosomula obtained from both protocols were indistinguishable from each other (Supplementary Video S1 and Video S2) and both schistosomula preparations progressed to later stages in the life cycle (up to two weeks post transformation) when cultured *in vitro* (data not shown). In terms of recovery, MT yields ∼90% of the applied cercariae, while the ST yields only ∼10%.

### Differential gene expression between MT and ST schistosomula

An overview of the sequencing and alignment results obtained for the sequenced samples is presented in Table 1; where samples 1 and 2 (for both MT and ST) represent independent schistosomula transformations (replicates). In the case of ST samples, and due to the limited number of schistosomula obtained in each experiment, approximately 3 experiments were pooled to provide enough biological material.

**Figure 3:**
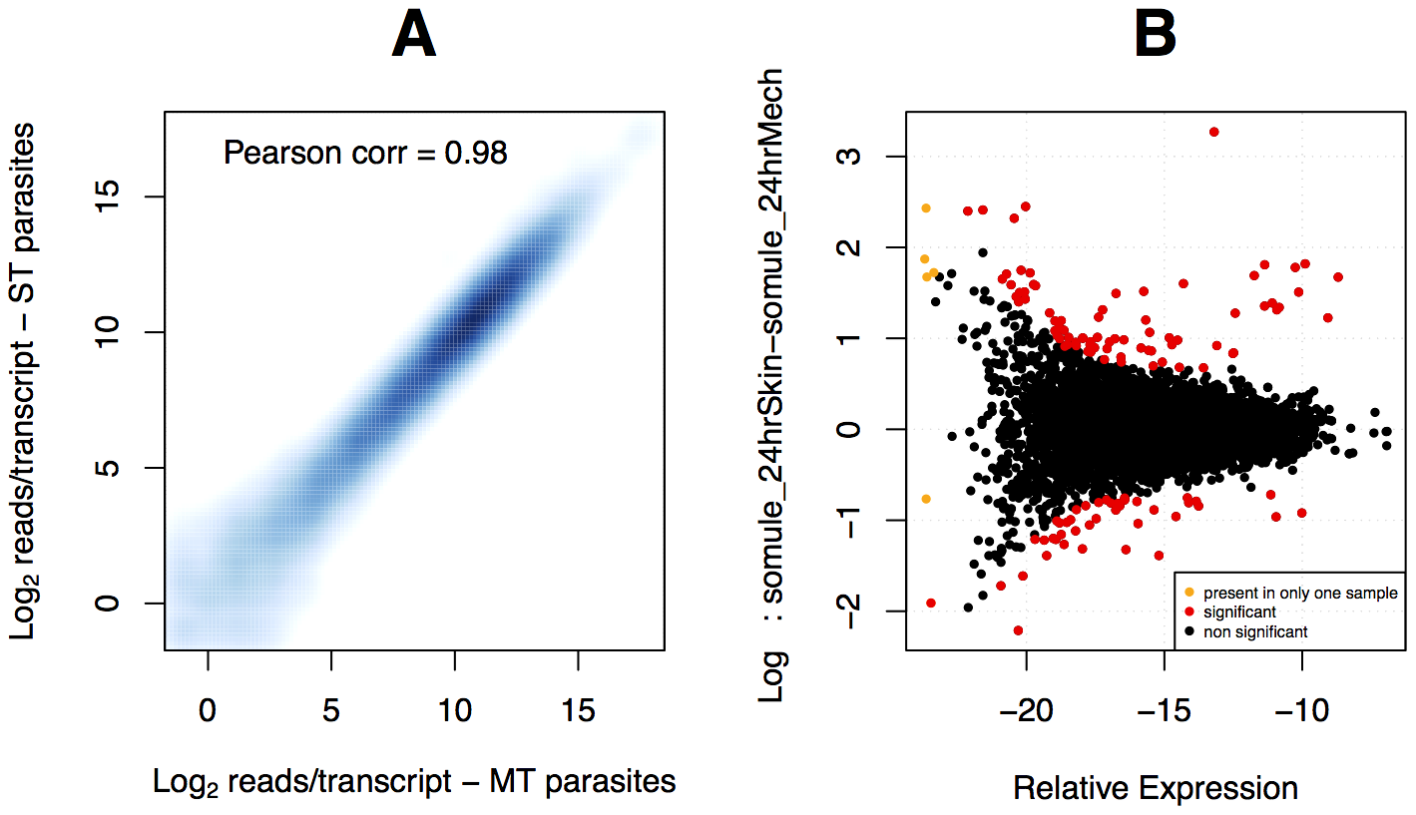
Skin-transformed (ST) and mechanically-transformed (MT) schistosomula are transcriptionally very similar. A – Correlation of gene expression values between MT (x-axis) and ST (y-axis) schistosomula. Both Pearson and Spearman's correlations are high (0.98 and 0.99 respectively) indicating very low variability between these two samples. B – Differential gene expression (MA plot) between MT and ST parasites at 24 hours after transformation (adjusted p-value<0.05). Relative concentration (x-axis) is plotted against fold change values (y-axis) in the log_2_ scale. Positive log_2_ fold changes represent transcripts more expressed in ST schistosomula while negative log_2_ fold changes represent transcripts more expressed in MT schistosomula.

Our dataset included 11,778 annotated transcripts and we found that 9,291 showed expression above background in at least one of the samples. Correlation analysis of the 24-hour old MT and ST schistosomula transcriptome samples showed high values for both Pearson's product and Spearman's rank coefficients (0.98 and 0.99, respectively; Figure 3A). Using the software package EdgeR [37], we found only 38 differentially expressed transcripts (adjusted p-value<0.05) between MT and ST schistosomula. Of these, 28 transcripts showed higher relative expression in the ST parasites (Table 2) while 10 transcripts showed higher relative expression in the MT (Table 3). A graphical representation of differentially expressed transcripts is shown in Figure 3B.

**Table 2:** Genes with higher expression in skin-transformed schistosomula.

Gene identifier	Fold change	Adj. P-value	Gene product description
Smp_900100	9.25	1.57E-32	NADH dehydrogenase subunit 3
Smp_204970	5.06	1.23E-03	Hypothetical protein
Smp_172770	4.76	6.73E-03	Hypothetical protein
Smp_900040	3.43	5.68E-18	NADH dehydrogenase subunit 2
Smp_900020	3.41	7.30E-18	NADH dehydrogenase subunit 6
Smp_900110	3.36	1.94E-17	NADH dehydrogenase subunit 1
Smp_029780	3.34	4.77E-02	Hypothetical protein
Smp_900060	3.16	9.52E-16	Cytochrome c oxidase subunit III
Smp_900050	3.14	1.39E-11	NADH dehydrogenase subunit 5
Smp_028850	3.03	1.50E-13	Hypothetical protein
Smp_127860	2.85	3.82E-04	Fmrfamide receptor
Smp_202120	2.85	1.05E-06	Hox class homeodomain protein djabd bb;
Smp_900090	2.79	1.33E-12	NADH dehydrogenase subunit 4
Smp_067800	2.64	9.45E-16	Fibrillin 2
Smp_900030	2.51	5.14E-10	ATP synthase F0 subunit 6
Smp_900070	2.5	7.99E-10	Cytochrome B
Smp_900010	2.45	1.94E-09	Cytochrome c oxidase subunit II
Smp_900080	2.39	3.71E-13	NADH dehydrogenase subunit 4L
Smp_170630	2.33	4.57E-02	Periostin, putative
Smp_146760	2.31	6.40E-04	Hypothetical protein
Smp_900000	2.3	3.92E-08	Cytochrome c oxidase subunit I
Smp_155320	1.97	1.43E-04	Hypothetical protein
Smp_212760	1.89	1.97E-06	Kinesin, putative
Smp_151600	1.87	2.00E-02	Neuronal calcium sensor 2
Smp_057860	1.78	3.08E-03	Hypothetical protein
Smp_132670	1.77	2.75E-03	Myosin regulatory light chain 2 smooth muscle
Smp_001070	1.59	5.51E-03	Hypothetical protein
Smp_211020	1.59	6.59E-03	Cell adhesion protein

Differentially expressed genes with adjusted p-value<0.05 are shown. Gene identification numbers with a prefix “Smp_90” are those encoded in the mitochondrial genome.

**Table 3:** Genes with higher expression in mechanically transformed schistosomula.

Gene identifier	Fold change	Adj. P-value	Gene product description
Smp_180340	6.06	2.35E-02	MEG-2 (ESP15) family
Smp_113660	4.63	4.57E-03	U1 small nuclear ribonucleoprotein C
Smp_147730	2.57	1.19E-05	Single kunitz protease inhibitor; serine type protease inhibitor
Smp_203400	2.45	1.77E-02	Rhodopsin orphan GPCR
Smp_204260	2.08	1.11E-02	Mastin
Smp_002150	1.93	2.86E-07	Mastin, subfamily S1A unassigned peptidase (S01 family)
Smp_089670	1.88	1.32E-06	Alpha 2 macroglobulin
Smp_063330	1.73	6.73E-03	Hypothetical protein
Smp_124000	1.60	2.84E-02	MEG-14
Smp_070240	1.54	1.08E-02	Venom allergen-like (VAL) 7 protein

Differentially expressed genes with adjusted p-value<0.05 are shown.

**Figure 4:**
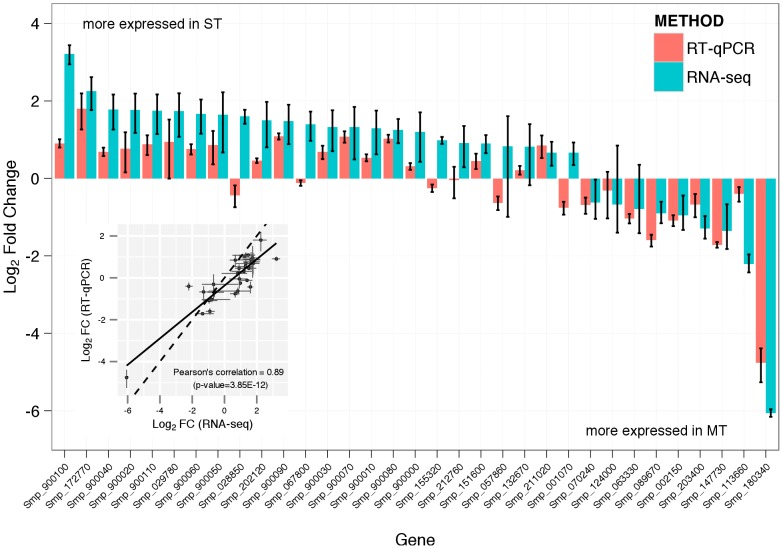
RT-qPCR and RNA-seq fold change values are highly correlated. Bar plots and inset scatter plot show a comparison of RNA-seq and RT-qPCR fold change values obtained for 33 differentially expressed genes. Fold change values are represented in the log_2_ scale and error bars represent 95% confidence interval (fold change ±1.96xSD). The inset scatter plot highlights the high correlation found between both methods (Pearson's correlation = 0.89, p-value = 3.85E^−12^). The solid lane represents the linear regression of this correlation; as a guide the x = y correlation is also shown as a dashed line.

RT-qPCR validation was performed for 33 of the 38 genes found differentially expressed between ST and MT schistosomula (Figure 4). With 95% confidence interval, the fold change values obtained from both methods overlapped in 14 cases (Smp_029780, Smp_057860, Smp_124000, Smp_132670, Smp_172770, Smp_211020, Smp_212760, Smp_900010, Smp_900020, Smp_900030, Smp_900050, Smp_900070, Smp_900080, Smp_900090). Moreover, fold change values obtained from these two different methods are highly correlated (Pearson's correlation 0.89, p-value 3.85E^−12^) and only in 4 cases (Smp_028850, Smp_067800, Smp_155320, Smp_001070) the direction of the fold change disagrees between the two methods.

### Genes more highly expressed in ST schistosomula

**Figure 5:**
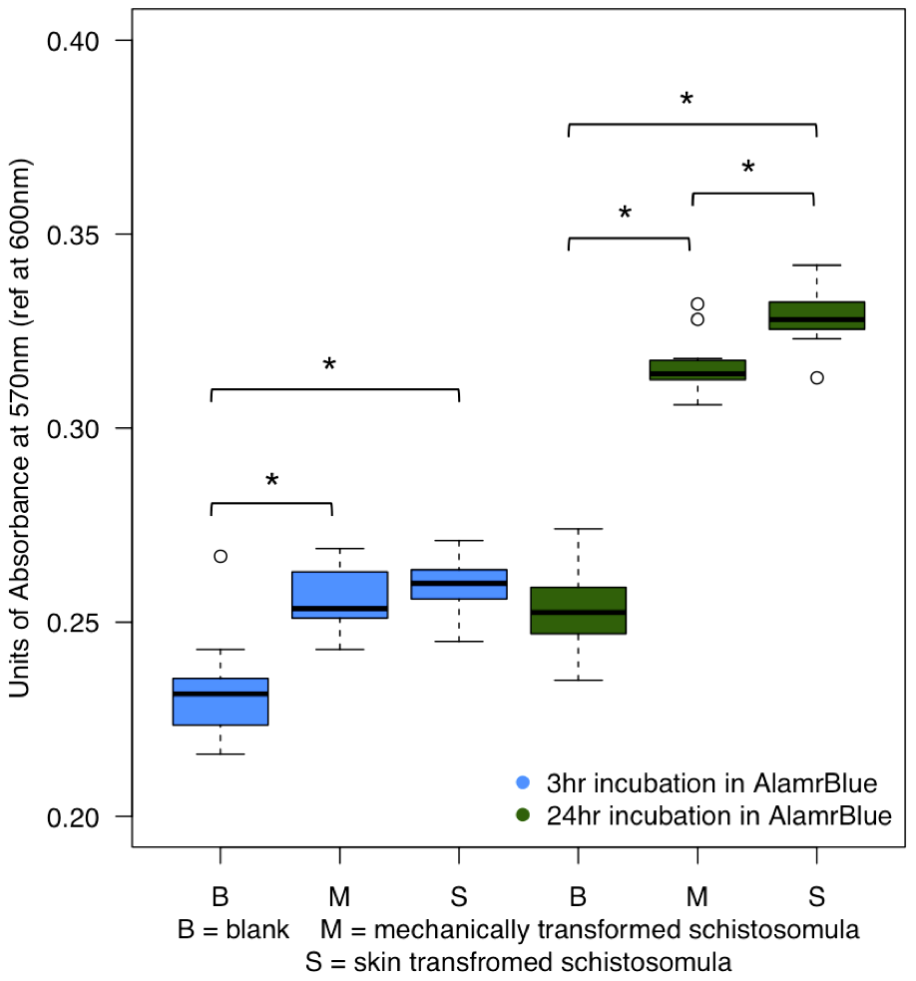
Mitochondrial activity is higher in ST schistosomula. AlamarBlue reactivity of 24-hour old MT and ST schistosomula incubated for either 3 hours (blue) or 24 hours (green). Longer incubation time in AlamarBlue provided the necessary resolution to establish a significant difference (*t-*test, p-value<0.01, indicated with * ) in metabolic activity between the mechanically (M) and skin (S) transformed schistosomula; (B) Blank wells.

Table 2 shows a list of genes more highly expressed in skin-transformed parasites. We found that all 12 mitochondrial genes (Smp_900000–Smp_900110) are found in this list. In order to investigate whether the higher expression of the mitochondrial genes had any consequences on metabolic activity we used the AlamarBlue (AB) assay. AB is a good indicator of mitochondrial activity through the measurement of redox species generated by the respiratory electron chain [42]. Incubation of 24-hour old schistosomula for 3 hours in AB showed no significant difference between MT and ST parasites (Figure 5 – blue boxplots). Increasing the incubation time to 24 hours showed an incremental increase in the absorbance (compared to blank wells) and a significant difference (*t*-test, p-value<0.01) between MT and ST schistosomula (Figure 5 – green boxplots) suggesting that these two populations of parasites have not only different rates of mitochondrial metabolism but that ST parasites are more metabolically active than their MT counterparts after 24 hours of *in vitro* culture.

The remainder of genes found relatively more expressed in the ST sample included examples that could be associated with the infection process. Two of these genes, for instance, are involved in calcium sensing (Smp_151600) or binding (Smp_132670), functions that have been associated with schistosomula adaptation to the mammalian host [43]. It is possible that such mechanisms are induced by contact with the host skin explaining the reduced expression of such transcripts in MT parasites.

### Genes more highly expressed in MT schistosomula

Proteases and proteases inhibitors are among the transcripts that were more expressed in MT schistosomula. Proteases have a recognised role in schistosomes; for example, adult worms use a set of aspartic proteases called cathepsins for the purpose of feeding [44] while cercariae use elastase and other proteases during the process of skin invasion [45], [46]. We found a gene encoding a secreted serine protease from the trypsin family (Smp_002150), with expression in MT parasites double that of ST parasites. RNA-seq data (Supplementary Table S3 in Ref. [32]) showed that the expression of this gene is developmentally up regulated after the transformation in schistosomula, showing an impressive 30-fold increase between 3-hour and 24-hour post-transformation parasites.

Alongside the serine protease we found two protease inhibitors that were also differentially expressed. Protease inhibitors can neutralise the action of host- and/or parasite-derived proteases. Smp_089670 encodes a 1,800 amino acids polypeptide with high similarity to an alpha-macroglobulin. Macroglobulin-type inhibitors entrap their target proteases limiting the range of substrates they can act upon; hence, they have a regulatory role rather than strictly inhibitory effect [47]. Macroglobulins can also inhibit coagulation [48], perhaps indicating that the secretion of the *S. mansoni* alpha-macroglobulin functions as a facilitator of schistosomula migration through the broken tissue/vessels during the skin stage. The second protease inhibitor was a kunitz-type serine protease inhibitor (Smp_147730). These types of inhibitors have been postulated to have an important role in the host-parasite interaction in *Echinococcus granulosus* infections [49]. Interestingly, both protease inhibitors were significantly up regulated during transformation from cercariae to schistosomula (Supplementary Table S3 in Ref. [32]) suggesting that their expression is developmentally regulated.

### Two microexon genes are more expressed in MT schistosomula

Microexons (<36 bp, in multiples of 3 bases) typically form a small part of some genes in most eukaryotes [50] but for a few genes in *S. mansoni*, microexons comprise the majority (∼75%) of the sequence length and these genes have therefore been termed microexon genes (MEGs) [51], [52]. Each MEG has the potential to generate an enormous repertoire of splicing variants through exon skipping because missed exons do not cause frame-shifts. The particular gene structure of MEGs therefore provides an easy mechanism to generate protein variation and seems to be both time and tissue specific [52].

**Figure 6:**
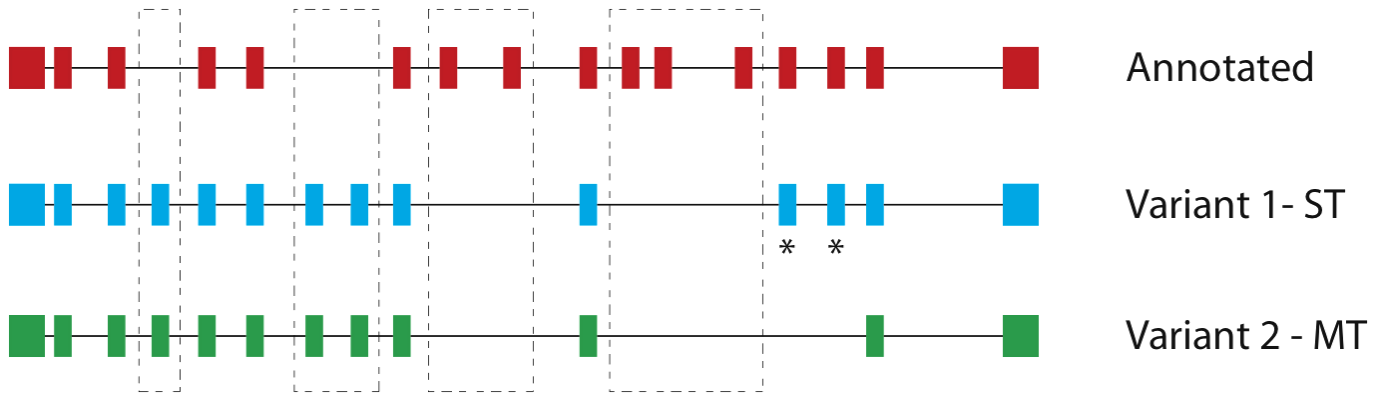
The microexon gene Smp_124000 is expressed as different isoforms in the ST and MT schistosomula. The exons or lack of them that contribute to the new isoform are marked in dashed boxes while exons that are differentially expressed between the ST and MT schistosomula are marked with a star (*).

Two MEGs from two different families appeared more expressed in MT compared to ST schistosomula at 24 hours after transformation (Table 3). In the case of Smp_180340, a MEG-2 member, RNA-seq coverage was poor and not specific to the exons in both samples; probably indicating unprocessed transcripts and was not considered for further analysis. For Smp_124000, RNA-seq data from schistosomula samples agreed with the current annotation of the gene (Figure 6). Intriguingly however, the isoforms expressed in MT and ST differed from each other, with three exons that were expressed in the ST being absent from the MT schistosomula sample (Figure 6). Because the RNA-seq experiments assayed the transcriptional status of large numbers of parasites simultaneously, we can therefore rule out – at least in this example – that exon skipping is simply a stochastic process.

## Discussion

The process of cercarial invasion and early stages of schistosomula migration are relevant for the development of intervention strategies against schistosomiasis. The skin schistosomula stage represents the first encounter of the parasite with the mammalian host and it is regarded as a vulnerable stage for parasite killing [53]–[55]. Hence, schistosomula have been the target of many studies that focus on both the adaptation of the parasite to its host and the identification of drug targets and vaccine development.

The study of changes in gene expression across different stages of host invasion can be used to investigate parasite adaptation to the host. With one exception, where *in vivo* recovered and *in vitro* cultured *S. japonicum* schistosomula were compared [56], all high-throughput gene expression studies have used MT schistosomula at different developmental stages by just prolonging the *in vitro* incubation time [25], [27], [28], [32], [57]. Since MT schistosomula are only proxies for natural infections, the differences between these and more naturally transformed parasites needs to be established. For instance, misleading artefactual parasite responses induced by stress or damage need to be identified as well as potentially important parasite responses that are only induced during the rapid natural transformation of free-living cercariae into obligatory parasitic schistosomula. Our work used the latest RNA-seq technology to investigate the differences in the gene expression of MT and ST schistosomula at 24-hour post-transformation. We found that these samples differ only in the expression of 38 transcripts (out of 9,291 expressed transcripts; adjusted p-value<0.05). In order to validate our approach, we performed RT-qPCR on 33 out of the 38 genes found differentially expressed and found that, at least in this experiment, RNA-seq seems to over estimate the fold change values of differentially expressed genes. However, a high correlation value of 0.89 was found between the two methods (similar values have been reported elsewhere [58]) suggesting that the RNA-seq is a valid and reliable method for high-throughput identification of differentially expressed genes. Increased transcript coverage (greater sequencing depth) as well as the addition of more biological replicates may result in better measurement by providing greater statistical power.

Transcripts encoded in the mitochondrial genome (mitochondrial genes) were found more highly expressed in ST parasites resulting in higher metabolic rates in ST parasites. Previously Brink *et al.*, [10] suggested that ST parasites are a selection of the most “fit” cercariae. We suggest that MT preparations may contain a mixture of fit and less-fit parasites and therefore not all of them are expected to develop at their maximum metabolic rate resulting in an averaged reduced metabolic activity for the MT schistosomula population.

MT parasites showed higher expression of a protease and two protease inhibitors. Proteases have been linked to host tissue invasion [59], [60] and have been shown expressed in parasites recovered from *in vivo* infections [56] as well as in purely MT schistosomula when compared to cercariae [28]. Therefore it is not surprising that they are present in our schistosomula samples. However, the unexpected finding is that these are more expressed in the MT rather than in the ST sample. If proteases were linked to the process of invasion, it would be expected that these be triggered by the presence of components of the skin, elements that are absent during the MT transformation. Further research will be needed to understand what triggers the expression of these genes.

Two members of the microexon gene family were found overexpressed in the MT parasites. What is more, the transcript variants found here are different from the ones previously reported confirming that different splice variants from the same loci are expressed at different time points of the life cycle. Interestingly, one of the variants expressed in 24 hours old schistosomula has a different exon profile in the two preparations, suggesting that cues from the environment might be triggering splicing variation. The role and regulation of alternate splicing in MEG may become clearer as the functions of microexon genes are further elucidated.

Due to the different treatments received by MT and ST schistosomula, including the low temperature and mechanical stress endured by MT schistosomula, we had hypothesised that stress-associated transcripts (e.g. stress/apoptotic pathways) would be differentially expressed. Surprisingly, we could not identify clear markers of stress, possibly because we had optimised our MT protocol to yield a minimum proportion of damaged parasites. Other MT protocols involving, for example a greater number of passages through a syringe-needle or a different source of mechanical stress may give different results.

Since MT schistosomula are not exposed to skin lipids that are known to play a role in transformation [18], [19] and induce the release of contents from the acetabular glands [20], we had anticipated observing differences related to the presence of lipids in the ST. However, we could not identify transcripts related to the binding (fatty-acid binding proteins) or to the transport of fatty acids and therefore conclude that the effect elicited by the presence of skin lipids is independent from transcriptional regulation at that time and is more likely related to machinery that the parasite may already have in place prior to its encounter with host skin.

Previously, gene expression changes using *in vivo* recovered (IVS) and mechanically transformed schistosomula (MTS) in *S. japonicum* at 3 days after transformation has been published [56]. The authors showed that IVS parasites show higher expression of transcripts encoding protaglandins, glutathione-S-transferase Sm28GST, paramyosin, stress related proteins and transcripts related to markers of anti-inflammatory and immunomodulatory processes. In the case of MTS parasites, the authors report higher expression of transcripts involved in glucose transport, fatty acids transport and haemoglobin digestion. None of the genes found differentially expressed in our analysis could be associated with the functions described in the *S. japonicum* study; probably due to the differences in the experimental design of both studies (i.e., time points at which differential expression is assessed).

Our study represents a snapshot of the schistosomula transcriptome after transformation. It is possible that a greater effect of the different treatments applied to both populations might be seen at shorter times after transformation. Ideally, a time-course experiment comprising more than one time point comparison between ST and MT schistosomula should have been performed. Nevertheless, should differences in gene expression exist at earlier time points, they disappear at 24 hours after transformation and are unlikely to have consequences on the gene expression profile of the parasites.

Finally, we emphasise that in view of the great differences in the transformation processes analysed here, the number of genes found differentially expressed between ST and MT at 24-hours post-transformation is unexpectedly small, suggesting that changes in gene expression induced upon transformation might be independent from the methodology employed to transformed parasites, at least in the two methods here studied.

In this work, we provide further evidence that transformation might be triggered by more robust signals, such as the change in osmotic pressure [17] and/or temperature [15] between the water and the host environments. We recommend that, except for the reported genes, these samples should be considered as transcriptionally equivalent. Our work contributes to the validation of gene expression studies that have used MT schistosomula and provides further evidence that the MT is a good proxy for natural skin-transformation.

## Supporting Information

Table S1 Complete list of differentially expressed genes. Fold change values, p-values and adjusted p-values are shown for each transcript as reported by EdgeR [37].
(XLS)

Table S2 Raw absorbance values collected for MT and ST samples during 3- and 24-hours incubation in Alamar Blue.
(XLS)

Video S1 This supporting information file shows schistosomula obtained using the optimised mechanical transformation protocol described in *Materials and Methods*.
(MP4)

Video S2 This supporting information file shows schistosomula obtained using the skin transformation [33].
(MP4)

## References

[1] Steinmann P, Keiser J, Bos R, Tanner M, Utzinger J (2006) Schistosomiasis and water resources development: systematic review, meta-analysis, and estimates of people at risk. Lancet Infect Dis 6: 411–425. 16790382 10.1016/S1473-3099(06)70521-7

[2] Ismail M, Metwally A, Farghaly A, Bruce J, Tao LF, et al (1996) Characterization of isolates of *Schistosoma mansoni* from Egyptian villagers that tolerate high doses of praziquantel. Am J Trop Med Hyg 55: 214–218. 8780463 10.4269/ajtmh.1996.55.214

[3] Melman SD, Steinauer ML, Cunningham C, Kubatko LS, Mwangi IN, et al (2009) Reduced susceptibility to praziquantel among naturally occurring Kenyan isolates of *Schistosoma mansoni* . PLoS Negl Trop Dis 3: e504. 19688043 10.1371/journal.pntd.0000504 PMC2721635

[4] Fallon PG, Doenhoff MJ (1994) Drug-resistant schistosomiasis: resistance to praziquantel and oxamniquine induced in *Schistosoma mansoni* in mice is drug specific. Am J Trop Med Hyg 51: 83–88. 8059919 10.4269/ajtmh.1994.51.83

[5] Ismail MM, Taha SA, Farghaly AM, el-Azony AS (1994) Laboratory induced resistance to praziquantel in experimental schistosomiasis. J Egypt Soc Parasitol 24: 685–695. 7844435

[6] Crabtree JE, Wilson RA (1980) *Schistosoma mansoni*: a scanning electron microscope study of the developing schistosomulum. Parasitology 81: 553–564. 7232034 10.1017/s003118200006193x

[7] Lawson JR, Wilson RA (1980) Metabolic changes associated with the migration of the schistosomulum of *Schistosoma mansoni* in the mammal host. Parasitology 81: 325–336. 7443296 10.1017/s0031182000056067

[8] Wilson RA, Lawson JR (1980) An examination of the skin phase of schistosome migration using a hamster cheek pouch preparation. Parasitology 80: 257–266. 7367041 10.1017/s0031182000000731

[9] Crabtree JE, Wilson RA (1985) *Schistosoma mansoni*: an ultrastructural examination of skin migration in the hamster cheek pouch. Parasitology 91 (Pt 1) 111–120. 4034241 10.1017/s0031182000056559

[10] Brink LH, McLaren DJ, Smithers SR (1977) *Schistosoma mansoni*: a comparative study of artificially transformed schistosomula and schistosomula recovered after cercarial penetration of isolated skin. Parasitology 74: 73–86. 320543 10.1017/s0031182000047545

[11] Samuelson JC, Caulfield JP (1985) The cercarial glycocalyx of *Schistosoma mansoni* . J Cell Biol 100: 1423–1434. 2985622 10.1083/jcb.100.5.1423 PMC2113874

[12] Stirewalt MA, Minnick DR, Fregeau WA (1966) Definition and collection in quantity of schistosomules of *Schistosoma mansoni* . Trans R Soc Trop Med Hyg 60: 352–360. 5919625 10.1016/0035-9203(66)90299-9

[13] Fishelson Z, Amiri P, Friend DS, Marikovsky M, Petitt M, et al (1992) *Schistosoma mansoni*: cell-specific expression and secretion of a serine protease during development of cercariae. Exp Parasitol 75: 87–98. 1639166 10.1016/0014-4894(92)90124-s

[14] Skelly PJ, Alan Wilson R (2006) Making sense of the schistosome surface. Adv Parasitol 63: 185–284. 17134654 10.1016/S0065-308X(06)63003-0

[15] Salafsky B, Fusco AC, Whitley K, Nowicki D, Ellenberger B (1988) *Schistosoma mansoni*: analysis of cercarial transformation methods. Exp Parasitol 67: 116–127. 2458958 10.1016/0014-4894(88)90014-8

[16] Coultas KA, Zhang SM (2012) In Vitro Cercariae Transformation: Comparison of Mechanical and Non-Mechanical Methods and Observation of Morphological Changes of Detached Cercariae Tails. J Parasitol 98: 1257–1261. 22519732 10.1645/GE-3072.1 PMC3638887

[17] Skelly PJ, Shoemaker CB (2000) Induction cues for tegument formation during the transformation of *Schistosoma mansoni* cercariae. Int J Parasitol 30: 625–631. 10779576 10.1016/s0020-7519(00)00031-x

[18] MacInnis AJ (1969) Identification of chemicals triggering cercarial penetration responses of *Schistosoma mansoni* . Nature 224: 1221–1222. 5358353 10.1038/2241221a0

[19] Austin FG, Stirewalt MA, Danziger RE (1972) *Schistosoma mansoni*: stimulatory effect of rat skin lipid fractions on cercarial penetration behavior. Exp Parasitol 31: 217–224. 5016593 10.1016/0014-4894(72)90112-9

[20] Stirewalt M (1978) Quantitative collection and proteolytic activity of preacetabular gland enzyme (s) of cercariae of *Schistosoma mansoni* . Am J Trop Med Hyg 27: 548–553. 677367 10.4269/ajtmh.1978.27.548

[21] Gazzinelli G, de Oliveira CC, Figueiredo EA, Pereira LH, Coelho PM, et al (1973) *Schistosoma mansoni*: biochemical evidence for morphogenetic change from cercaria to schistosomule. Exp Parasitol 34: 181–188. 4744838 10.1016/0014-4894(73)90077-5

[22] Howells RE, Ramalho-Pinto FJ, Gazzinelli G, de Oliveira CC, Figueiredo EA, et al (1974) *Schistosoma mansoni*: mechanism of cercarial tail loss and its significance to host penetration. Exp Parasitol 36: 373–385. 4214711 10.1016/0014-4894(74)90077-0

[23] Ramalho-Pinto FJ, Gazzinelli G, Howells RE, Mota-Santos TA, Figueiredo EA, et al (1974) *Schistosoma mansoni*: defined system for stepwise transformation of cercaria to schistosomule in vitro. Exp Parasitol 36: 360–372. 4139038 10.1016/0014-4894(74)90076-9

[24] Milligan JN, Jolly ER (2011) Cercarial transformation and in vitro cultivation of *Schistosoma mansoni* schistosomules. J Vis Exp DOI: 10.3791/3191. 10.3791/3191 PMC3217644 21876520

[25] Dillon GP, Feltwell T, Skelton JP, Ashton PD, Coulson PS, et al (2006) Microarray analysis identifies genes preferentially expressed in the lung schistosomulum of *Schistosoma mansoni* . Int J Parasitol 36: 1–8. 16359678 10.1016/j.ijpara.2005.10.008

[26] Farias LP, Tararam CA, Miyasato PA, Nishiyama MY Jr, Oliveira KC, et al (2011) Screening the *Schistosoma mansoni* transcriptome for genes differentially expressed in the schistosomulum stage in search for vaccine candidates. Parasitol Res 108: 123–135. 20852890 10.1007/s00436-010-2045-1

[27] Fitzpatrick JM, Peak E, Perally S, Chalmers IW, Barrett J, et al (2009) Anti-schistosomal intervention targets identified by lifecycle transcriptomic analyses. PLoS Negl Trop Dis 3: e543. 19885392 10.1371/journal.pntd.0000543 PMC2764848

[28] Gobert GN, Tran MH, Moertel L, Mulvenna J, Jones MK, et al (2010) Transcriptional changes in *Schistosoma mansoni* during early schistosomula development and in the presence of erythrocytes. PLoS Negl Trop Dis 4: e600. 20161728 10.1371/journal.pntd.0000600 PMC2817720

[29] Verjovski-Almeida S, DeMarco R, Martins EA, Guimaraes PE, Ojopi EP, et al (2003) Transcriptome analysis of the acoelomate human parasite *Schistosoma mansoni* . Nat Genet 35: 148–157. 12973350 10.1038/ng1237

[30] Abdulla MH, Ruelas DS, Wolff B, Snedecor J, Lim KC, et al (2009) Drug discovery for schistosomiasis: hit and lead compounds identified in a library of known drugs by medium-throughput phenotypic screening. PLoS Negl Trop Dis 3: e478. 19597541 10.1371/journal.pntd.0000478 PMC2702839

[31] Mortazavi A, Williams BA, McCue K, Schaeffer L, Wold B (2008) Mapping and quantifying mammalian transcriptomes by RNA-Seq. Nat Methods 5: 621–628. 18516045 10.1038/nmeth.1226 PMC13303166

[32] Protasio AV, Tsai IJ, Babbage A, Nichol S, Hunt M, et al (2012) A Systematically Improved High Quality Genome and Transcriptome of the Human Blood Fluke *Schistosoma mansoni* . PLoS Negl Trop Dis 6: e1455. 22253936 10.1371/journal.pntd.0001455 PMC3254664

[33] Clegg JA, Smithers SR (1972) The effects of immune rhesus monkey serum on schistosomula of *Schistosoma mansoni* during cultivation in vitro. Int J Parasitol 2: 79–98. 4631607 10.1016/0020-7519(72)90036-7

[34] Mansour NR, Bickle QD (2010) Comparison of microscopy and Alamar blue reduction in a larval based assay for schistosome drug screening. PLoS Negl Trop Dis 4: e795. 20706580 10.1371/journal.pntd.0000795 PMC2919390

[35] Trapnell C, Pachter L, Salzberg SL (2009) TopHat: discovering splice junctions with RNA-Seq. Bioinformatics 25: 1105–1111. 19289445 10.1093/bioinformatics/btp120 PMC2672628

[36] Quinlan AR, Hall IM (2010) BEDTools: a flexible suite of utilities for comparing genomic features. Bioinformatics 26: 841–842. 20110278 10.1093/bioinformatics/btq033 PMC2832824

[37] Robinson MD, Oshlack A (2010) A scaling normalization method for differential expression analysis of RNA-seq data. Genome Biol 11: R25. 20196867 10.1186/gb-2010-11-3-r25 PMC2864565

[38] Benjamini Y, Drai D, Elmer G, Kafkafi N, Golani I (2001) Controlling the false discovery rate in behavior genetics research. Behav Brain Res 125: 279–284. 11682119 10.1016/s0166-4328(01)00297-2

[39] Untergasser A, Nijveen H, Rao X, Bisseling T, Geurts R, et al (2007) Primer3Plus, an enhanced web interface to Primer3. Nucleic Acids Res 35: W71–74. 17485472 10.1093/nar/gkm306 PMC1933133

[40] Pfaffl MW (2001) A new mathematical model for relative quantification in real-time RT-PCR. Nucleic Acids Res 29: e45. 11328886 10.1093/nar/29.9.e45 PMC55695

[41] Busby MA, Gray JM, Costa AM, Stewart C, Stromberg MP, et al (2011) Expression divergence measured by transcriptome sequencing of four yeast species. BMC Genomics 12: 635. 22206443 10.1186/1471-2164-12-635 PMC3296765

[42] Springer JE, Azbill RD, Carlson SL (1998) A rapid and sensitive assay for measuring mitochondrial metabolic activity in isolated neural tissue. Brain Res Brain Res Protoc 2: 259–263. 9630663 10.1016/s1385-299x(97)00045-7

[43] Kusel JR, Al-Adhami BH, Doenhoff MJ (2007) The schistosome in the mammalian host: understanding the mechanisms of adaptation. Parasitology 134: 1477–1526. 17572930 10.1017/S0031182007002971

[44] Brinkworth RI, Prociv P, Loukas A, Brindley PJ (2001) Hemoglobin-degrading, aspartic proteases of blood-feeding parasites: substrate specificity revealed by homology models. J Biol Chem 276: 38844–38851. 11495896 10.1074/jbc.M101934200

[45] McKerrow JH (2003) Invasion of skin by schistosome cercariae: some neglected facts: Response from James J. McKerrow. Trends Parasitol 18: 66–68. 10.1016/s1471-4922(02)00019-3 12586470

[46] McKerrow JH, Salter J (2002) Invasion of skin by Schistosoma cercariae. Trends Parasitol 18: 193–195. 11983589 10.1016/s1471-4922(02)02309-7

[47] Armstrong PB (2006) Proteases and protease inhibitors: a balance of activities in host-pathogen interaction. Immunobiology 211: 263–281. 16697919 10.1016/j.imbio.2006.01.002

[48] de Boer JP, Creasey AA, Chang A, Abbink JJ, Roem D, et al (1993) Alpha-2-macroglobulin functions as an inhibitor of fibrinolytic, clotting, and neutrophilic proteinases in sepsis: studies using a baboon model. Infect Immun 61: 5035–5043. 7693593 10.1128/iai.61.12.5035-5043.1993 PMC281280

[49] Gonzalez S, Flo M, Margenat M, Duran R, Gonzalez-Sapienza G, et al (2009) A family of diverse Kunitz inhibitors from *Echinococcus granulosus* potentially involved in host-parasite cross-talk. PLoS One 4: e7009. 19759914 10.1371/journal.pone.0007009 PMC2740865

[50] Volfovsky N, Haas BJ, Salzberg SL (2003) Computational discovery of internal micro-exons. Genome Res 13: 1216–1221. 12799353 10.1101/gr.677503 PMC403649

[51] Berriman M, Haas BJ, LoVerde PT, Wilson RA, Dillon GP, et al (2009) The genome of the blood fluke *Schistosoma mansoni* . Nature 460: 352–358. 19606141 10.1038/nature08160 PMC2756445

[52] DeMarco R, Mathieson W, Manuel SJ, Dillon GP, Curwen RS, et al (2010) Protein variation in blood-dwelling schistosome worms generated by differential splicing of micro-exon gene transcripts. Genome Res 20: 1112–1121. 20606017 10.1101/gr.100099.109 PMC2909574

[53] Capron M, Capron A (1986) Rats, mice and men - models for immune effector mechanisms against schistosomiasis. Parasitol Today 2: 69–75. 15462774 10.1016/0169-4758(86)90158-4

[54] Loverde PT (1998) Do antioxidants play a role in schistosome host-parasite interactions? Parasitol Today 14: 284–289. 17040785 10.1016/s0169-4758(98)01261-7

[55] Wilson RA, Coulson PS (2009) Immune effector mechanisms against schistosomiasis: looking for a chink in the parasite's armour. Trends Parasitol 25: 423–431. 19717340 10.1016/j.pt.2009.05.011 PMC3686490

[56] Chai M, McManus DP, McInnes R, Moertel L, Tran M, et al (2006) Transcriptome profiling of lung schistosomula,in vitro cultured schistosomula and adult *Schistosoma japonicum* . Cell Mol Life Sci 63: 919–929. 16570121 10.1007/s00018-005-5578-1 PMC11136126

[57] Parker-Manuel SJ, Ivens AC, Dillon GP, Wilson RA (2011) Gene Expression Patterns in Larval *Schistosoma mansoni* Associated with Infection of the Mammalian Host. PLoS Negl Trop Dis 5: e1274. 21912711 10.1371/journal.pntd.0001274 PMC3166049

[58] Ramayo-Caldas Y, Mach N, Esteve-Codina A, Corominas J, Castello A, et al (2012) Liver transcriptome profile in pigs with extreme phenotypes of intramuscular fatty acid composition. BMC Genomics 13: 547. 23051667 10.1186/1471-2164-13-547 PMC3478172

[59] Hansell E, Braschi S, Medzihradszky KF, Sajid M, Debnath M, et al (2008) Proteomic analysis of skin invasion by blood fluke larvae. PLoS Negl Trop Dis 2: e262. 18629379 10.1371/journal.pntd.0000262 PMC2467291

[60] Salter JP, Lim KC, Hansell E, Hsieh I, McKerrow JH (2000) Schistosome invasion of human skin and degradation of dermal elastin are mediated by a single serine protease. J Biol Chem 275: 38667–38673. 10993899 10.1074/jbc.M006997200

